# The Benefits and Challenges of Virtual Education for Interprofessional Teams in a Post-COVID Environment

**DOI:** 10.3390/healthcare10112195

**Published:** 2022-11-02

**Authors:** Tiffany Champagne-Langabeer

**Affiliations:** School of Biomedical Informatics, University of Texas Health Science Center at Houston, 7000 Fannin St., Houston, TX 77030, USA; tiffany.champagne@uth.tmc.edu

There have been a series of disruptions in the healthcare environment since 2019, starting with the global pandemic. As we witnessed additional restrictions placed on visitors within hospital systems, we also saw similar restrictions placed upon the students who train in those hospitals [1]. In a matter of days, the academic community charged with educating future doctors, nurses, dentists, therapists, allied professionals, and non-clinical positions (such as informaticians) were faced with few options for satisfying their required training components. As the pandemic lingered, hospitals became overwhelmed, and licensed professionals were stretched beyond capacity. The healthcare community went into action, and policymakers quickly expedited rules surrounding telemedicine across state lines, early licensure for certain health professions, and opening various electronic platforms for physicians to communicate with their patients [2].

Virtual training programs also flourished during this time to prepare students to become practitioners in a condensed timeline. Before 2019, one study estimated that 85% of faculty at research institutions in the United States (U.S.) had never taught a class online [3]. As of 2022, most if not all courses taught in the U.S. have some form of online, asynchronous, or hybrid learning approach; further, the pandemic affected more than 90% of learners around the world in some way [4]. Many programs transitioned their didactic content entirely online and required students to learn in a flipped classroom approach. Flipped learning requires that the students go through the material and lessons in their own time and ask their instructor questions after. Flipped classrooms are typically asynchronous, but some have online synchronous components such as regular meetings through videoconference. While this approach is not new for higher education at large, it is a seismic shift for many in the healthcare field who are used to delivering content in large lecture halls or in clinical (in-person) environments. Flipped learning, however, has become the norm in many settings and can increase student engagement and the efficiency of both the student and instructor [5,6]. In 2021, the academic community of clinicians were often needed to work shifts in hospitals and clinics due to extreme shortages. For these clinicians, flipped classrooms and online learning offered an opportunity to use their limited face-to-face time working directly with students and deliver didactic content asynchronously and outside of a traditional in-person classroom.

Like online education, virtual care for patients is not a new concept. Innovative treatments using “interactive-television” were successfully used almost 30 years ago in psychiatric settings [7]. Further, audio-only telemedicine was used as a proxy for primary physician office visits as far back as 1879 as reported in the *Lancet* [8]. Despite the ubiquity of telemedicine in some settings such as military medicine, and the innovations in other settings such as in prehospital care, there were clinicians who were overwhelmed and hesitant to embrace online learning [9]. However, as the benefits of virtual care such as social distancing became more apparent to patients and clinicians, the healthcare industry saw this mindset shift in most clinicians. Areas such as teledentistry, once considered questionable for videoconferencing, were expanded in new and beneficial ways for patients [10], and processes once considered sacred in-person events, such as residency interviews, transitioned to virtual platforms with success [11]. On balance, there have been programmatic challenges, and the transition to virtual care and education has exposed the chasm in access to care and technology and training faced by many patients and students [12]. The effectiveness of virtual education in healthcare and patient satisfaction in receiving care can only be accomplished when all members have access to technology, stable internet, or a cellular network. Poverty is often considered the “biology of disadvantage” in public health settings, and this was evidenced when adopting and transitioning in-person curriculum to online settings while supporting students with decreased access to technology [13,14]. This remains a challenge for educators that must be solved.

The global outbreak of COVID-19 brought considerable disruptions to interprofessional teams that care for patients, and health educators faced new challenges when confronted with instructing students to care for patients in the virtual setting. In this issue of *Healthcare*, we will discuss recent successes and failures in the implementation of new models for interprofessional care within the virtual setting. The current Special Issue, “Innovations in Interprofessional Care and Training”, is dedicated to examining these trends, developing solutions, and supporting clinicians and healthcare educators in addressing current issues in interdisciplinary care within the recent context of delivering care virtually.
